# Downregulation of CyclophilinA/CD147 Axis Induces Cell Apoptosis and Inhibits Glioma Aggressiveness

**DOI:** 10.1155/2020/7035847

**Published:** 2020-07-24

**Authors:** Shengchao Xu, Chi Hu, Zhiqiang Xiao, Chengke Luo, Zhixiong Liu

**Affiliations:** ^1^Department of Neurosurgery, Xiangya Hospital of Central South University, Changsha, Hunan, China; ^2^Department of Neurosurgery, The First Affiliated Hospital, College of Medicine, Zhejiang University, Hangzhou, Zhejiang, China; ^3^Research Center of Carcinogenesis and Targeted Therapy, Xiangya Hospital of Central South University, Changsha, Hunan, China

## Abstract

Gliomas are the most common primary tumors in the brain with poor prognosis. Previous studies have detected high expression of Cyclophilin A (CyPA) and CD147, respectively, in glioma. However, the correlation between their expressions and glioma prognosis remains unclear. Here, we investigated the expression of CyPA and CD147 in different types of glioma and characterized their relationships with clinical features, prognosis, and cell proliferation. Results showed that CyPA and CD147 expressions were elevated in higher grade gliomas. Moreover, the knockdown of CyPA and CD147 by RNA interference significantly induced cell express apoptosis biomarkers such as Annexin V and inhibited proliferation biomarkers like EdU in glioma cells. In summary, our findings revealed that high expression of CyPA and CD147 correlated with glioma grades. Moreover, downregulation of the Cyclophilin A/CD147 axis induces cell apoptosis and inhibits glioma aggressiveness. Those indicating CyPA and CD147 could be used as both potential predictive biomarkers and a potential therapeutic target.

## 1. Introduction

Gliomas are the most common primary tumors in the brain with a prevalence of between 5 and 10 cases per 100,000 people, accounting for 81% of central nervous system malignancies [1]. Histologically, gliomas are divided into four malignancy grades based on the World Health Organization classification [2], with prognosis dependent on tumor grade and histology. However, despite considerable therapeutic efforts, glioblastoma (GBM) remains the most common form of human malignant brain tumor, whereas the overall 5-year survival rate of GBM is less than 5% and even worse in elderly patients. It is necessary to identify valid biomarkers to accurately predict the prognosis of glioma patients.

Cyclophilin A (CyPA), also known as peptidylprolyl isomerase A (PPIA), is an enzyme encoded by the *PPIA* gene on chromosome 7. It is a member of the immunophilin family that belongs to the peptidyl-prolyl isomerase family. Proteins in this family catalyze cis-trans isomerization of peptidyl-prolyl bonds that precede the proline amino acid [3]. CyPA binds with membrane receptor or intracellular partners, subsequently activating the downstream signaling pathway. In the nucleus, the localization of CyPA is observed in serine protease-dependent microtubule intervention, indicating that CyPA is associated with cell cycle [4]. Moreover, CyPA can be secreted out of cells. With the presence of reactive oxygen species, cells secrete CyPA to induce an inflammatory response and alleviate tissue injury [5]. Therefore, CyPA is found to be involved in inflammatory diseases and autoimmune diseases [6]. Besides, previous studies have demonstrated that extracellular CyPA promotes tumor proliferation, migration, and drug resistance in various studies [7–9].

CD147, encoded by the BSG gene, is a member of the immunoglobulin superfamily binding with cell membrane. As a type I integral membrane receptor, it contains 269 amino acids that form two heavily immunoglobulin-like domains at N-terminal [10]. CD147 was found on the surface of tumor cells and may trigger the production or release of matrix metalloproteinase (MMP) in surrounding mesenchymal and tumor cells, thereby contributing to tumor invasion [11]. Immunomodulatory drugs such as thalidomide were found to treat multiple myeloma by suppressing the stabilization of CD147 complex. Besides, elevated expression of CD147 is associated with the efficacy of the treatment [12]. In addition, CD147 was associated with epithelial to mesenchymal transition (EMT) in prostate cancer and indicate poor survival rate [13]. Moreover, CD147 could protect malignant melanoma cells from hydrogen peroxide-induced oxidative stress [14].

As a membrane receptor, CD147 acts as the primary signaling receptor of extracellular CyPA [15]. The interaction of CyPA and CD147 was found to promote the proliferation and bone marrow homing of multiple myeloma cell [16]. However, the role of CyPA and CD147 in glioma remains unknown. In this study, we found that both CyPA and CD147 are highly expressed in higher grade glioma compared with lower grade. Considering coexpression of CyPA and CD147 axis and the limitation of single-biomarker prediction in cancer prognosis, we expected CyPA/CD147 axis could be not only promising potential biomarkers in glioma prognosis and liquid biopsy but also a potential therapeutic target.

## 2. Materials and Methods

### 2.1. Ethics Approval and Consent to Participate

This study was approved by the ethics committee of Xiangya Hospital, Central South University (CSU; Changsha, China), and written informed consent was obtained from all patients.

### 2.2. Patient Tissue Specimens

Samples of glioma and nontumor tissues were collected from 21 patients at the Department of neurosurgery, Xiangya Hospital, Central South University. Patients with malignant peripheral nerve sheath tumors or other unrelated neurologic tumors were excluded. The tumor tissues were collected from the edge of glioma lesions and confirmed by at least 2 experienced pathologists. Specimens were rapidly frozen in liquid nitrogen and immediately transferred to a −80°C freezer immediately for subsequent experiments. The clinical features of all patients were collected and analyzed.

### 2.3. TCGA and CGGA Data Analysis

For gene expression analysis, the expression counts from The Cancer Genome Atlas (TCGA) glioma RNA-seq datasets were downloaded from GDC Data Portal, https://portal.gdc.cancer.gov, including 5 normal brain tissue, 530 LGG, and 169 GBM samples. The expression counts from the Chinese Glioma Genome Atlas (CGGA) RNA-seq datasets were downloaded from the website, http://cgga.org.cn, including 182 LGGs and 139 GBMs [17, 18]. The expressions of CyPA and CD147 from RNA-seq data were clustered and visualized with the GEPIA website tool, http://gepia.cancer-pku.cn [19], GraphPad Prism v.5.0 (GraphPad Software, Inc., La Jolla, CA, USA), and SPSS v.16 (SPSS, Inc., Chicago, IL, USA).

### 2.4. Immunohistochemistry

Immunohistochemistry was performed in 4 *μ*m paraffin sections. The tissue sections were dewaxed in xylene, rehydrated in graded ethanol, and rinsed in distilled water. Citrate buffer (pH = 6.0) was used to retrive the antigen, and 0.3% H_2_O_2_ was used to block endogenous peroxidase activity. After the blockade of nonspecific binding, tissue sections were incubated with the Cyclophilin A antibody (cat. no. 5360s; 1 : 150 dilution; Santa Cruz Biotechnology, Inc.) and CD147 antibody (cat. no. 13287; 1 : 400 dilution; Cell Signaling Technology, Inc.) for overnight at 4 centigrade. The secondary antibody was applied using the Envision Detection kit (Dako; Agilent Technologies, Inc., Santa Clara, CA, USA). The staining was performed by the application of diaminobenzidine tetrahydrochloride (DAB) for 2 min and hematoxylin for 1 min at room temperature. The stained sections were evaluated with a Nikon microscope in 10 independent fields at magnification, ×400. We followed the methods of Mu et al. [20].

### 2.5. Cell Culture and Transfection

Human glioma cell lines including U251, U87, U343, SHG44, HS683, and HEB were purchased from the American Type Culture Collection (Manassas, VA, USA). All cells were cultured in DMEM medium with 10% fetal bovine serum (Gemini Bio Products, West Sacramento, CA, USA) at 37°C in a humidified atmosphere containing 5% CO_2_. Cells were transfected with two siRNAs targeting at CyPA and CD147 (CD147 target sequence: 5′-GAA GTC GTC AGA ACA CAT CAA CG-3′and 5′-TTC CGG CGC TTC TCG TAG A-3′; CyPA target sequence: 5′-CCC ACC GTG TTC GAC ATT-3′ and 5′-GGA CCC GTA TGC TTTA GGA TGA-3′) synthesized by Guangzhou RiboBio Co., Ltd., (Guangzhou, China). The cell transfections were performed using lipofectamine max (Invitrogen; Thermo Fisher Scientific, Inc., Waltham, MA, USA) according to the manufacturer's protocol. The cells were collected after 48-hour transfection. We followed the methods of Mu et al. [20].

### 2.6. RNA Extraction and RT-qPCR Analysis

Total RNA was extracted from cells using the TriPure Isolation reagent (Invitrogen, USA). cDNA was synthesized using the Transcription First Strand cDNA Synthesis kit (Promega, USA). GAPDH was used as the reference. The primer sequences are as follows: CyPA forward, 5′-CAG GGA GTA CGT GCG GGT GT-3′, CyPA reverse, 5′-TCG GTC GCC GCT TCC CAG TT-3′; CD147 forward, 5′-GAG AGC AGG TTC TTC GTG AGT TC-3′, CD147 reverse 5′-GCC TTT GTC ATT CTG GTG CTG-3′; GAPDH forward, 5′-AAG TGA AGC AGG AGG GTG GAA-3′, GAPDH reverse, 5′-CAG CCT CAC CCC ATT TGA TG-3′. The quantification of RNA expressions was performed using SYBR Green qPCR Mix (GeneCopoeia, USA). The results were normalized using the 2^-*ΔΔ*Ct^ method. We followed the methods of Mu et al. [20].

### 2.7. Western Blot Analysis

Total proteins were obtained using RIPA buffer with protease inhibitor and phosphatase inhibitor. The concentration of the protein was determined by the BCA Protein Assay kit (Pierce; Thermo Fisher Scientific, Inc.). After the separation in 10% SDS-PAGE, proteins were transferred to PVDF membrane and incubated with skim milk for 1 hour at room temperature. The primary antibodies used were as follows: Cyclophilin A (cat. no. 5360), CD147 (cat. no. 13287) (all from Cell Signaling Technology, Inc., Danvers, MA, USA, and used at a dilution of 1 : 1,000) and *β*-actin (cat. no. SC-130300, Santa Cruz Biotechnology, Inc., Dallas, TX, USA). After the incubation with primary antibody overnight at 4°C, HRP-labeled secondary antibody was applied for 1 h at room temperature. The protein was visualized using ECL reagent. We followed the methods of Mu et al. [20].

### 2.8. 5-Ethynyl-2′-Deoxyuridine (EdU) Assay

Cell proliferation was detected by an EdU Cell Proliferation Assay Kit (Ribobio, Guangzhou, China) according to the manufacturer's instructions. The proportion of cells that incorporated EdU was determined with a fluorescence microscope (Nikon C2, Tokyo, Japan).

### 2.9. Flow Cytometry Analysis

A total of 1 × 10^6^ cells were resuspended in a single cell suspension and washed two times with PBS solution. The cell apoptosis analysis was performed with the Annexin V-FITC/PI Apoptosis Detection Kit (BD Biosciences, USA) according to the manufacturer's instructions. The ratio of alive cells was detected by flow cytometry (BD FACSCanto II, USA).

### 2.10. Statistical Analysis

All statistical analyses were performed using GraphPad Prism v.5.0 (GraphPad Software, Inc., La Jolla, CA, USA) and SPSS v.16 (SPSS, Inc., Chicago, IL, USA). These experiments were performed independently at least three times. Student's *t*-test was used to compare the differences between two groups. One-way ANOVA was used to assess the differences between three or more groups. The Mann–Whitney *U* test was performed to determine the expression of CyPA and CD147 proteins among normal, LGG, and GBM samples. CyPA and CD147 expression and its correlation with clinical features were calculated by the *χ*^2^ test. All ROC curve analyses were performed using SPSS v.16 (SPSS, Inc., Chicago, IL, USA). Different grades of glioma were set as status variables, whereas expression counts of CyPA and CD147 were set as test variables. For survival analysis, groups were divided based on the cut-off points determined by the X-tile tool [21]. Survival curves were established using the Kaplan–Meier method and compared by the log-rank test. *P* < 0.05 was considered statistically significant.

## 3. Results

### 3.1. High Expression of CyPA and CD147 Is Associated with Glioma Grade, Histological Type, and Prognosis

**Figure 1:**
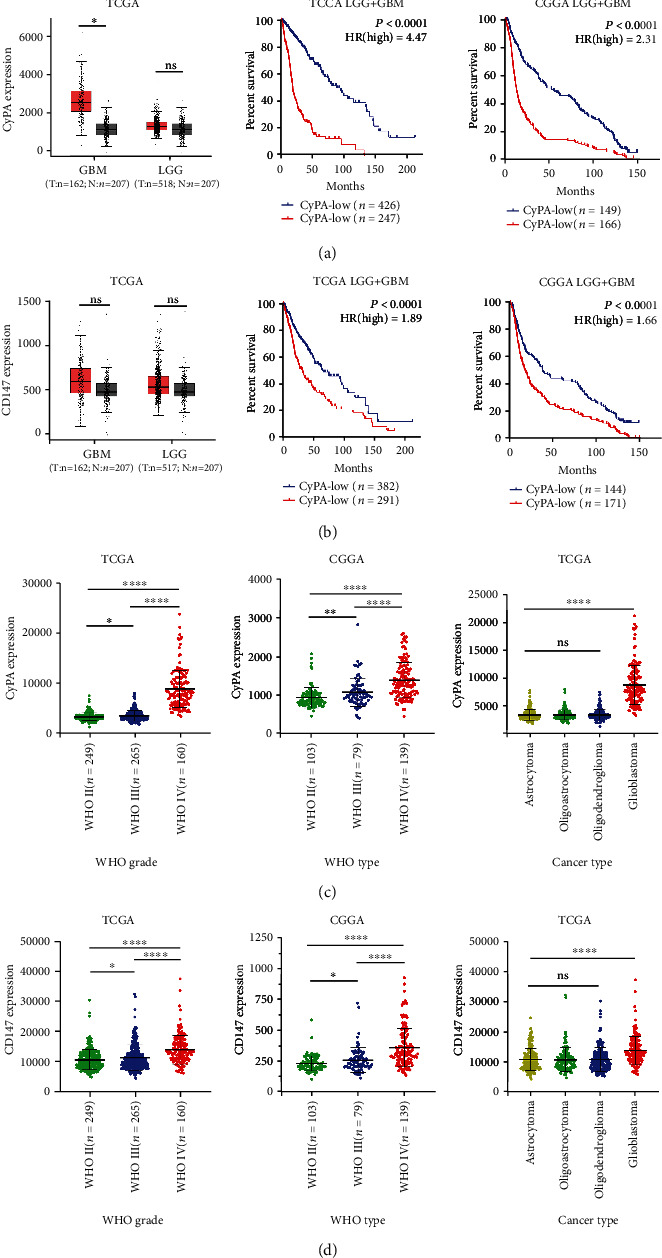
Expressions of CyPA and CD147 are associated with glioma grade, histological type, and prognosis. The expression of CyPA (a) and CD147 (b) in GBM and LGG compared with normal tissue and correlation with prognosis in TCGA and CGGA databases. The expression of CyPA (c) and CD147 (d) in different WHO grades and histological types of glioma in TCGA and CGGA database. ^∗^*P* < 0.05, ^∗∗^*P* < 0.01, ^∗∗∗∗^*P* < 0.0001, ns stands for no significance.

In order to explore the role of CyPA and CD147 in glioma, we collect and analyze the mRNA expression and clinical information of patients with glioma from TCGA and CGGA databases. Results show that CyPA is highly expressed in GBM compared with normal brain tissue whereas there is no significant difference between low-grade glioma (LGG) and normal brain tissue. However, the high expression of CyPA is associated with poor prognosis (Figure 1(a)). As for CD147, no significant difference was found between glioma and normal brain tissue. Nevertheless, the high expression of CD147 predicts poor prognosis (Figure 1(b)). Further, we screen the expression of CyPA and CD147 in different grades and histological types of glioma. Results show that the expression of CyPA and CD147 is elevated in higher grade glioma. No significant difference is found among astrocytoma, oligoastrocytoma, and oligodendroglioma, whereas CyPA and CD147 are highly expressed in glioblastoma compared with other histological types (Figures 1(c) and 1(d)). We further validate the expression of CyPA and CD147 in clinical specimens (Table S1). The expression of CyPA and CD147 is higher in glioblastoma compared with nonneoplastic and diffuse astrocytoma (Figure S1A-B). However, no significant difference was detected between nonneoplastic and diffuse astrocytoma (Figure S1C-D).

### 3.2. Expression of CyPA and CD147 Could Be Inhibited by Specific Si-RNA

**Figure 2:**
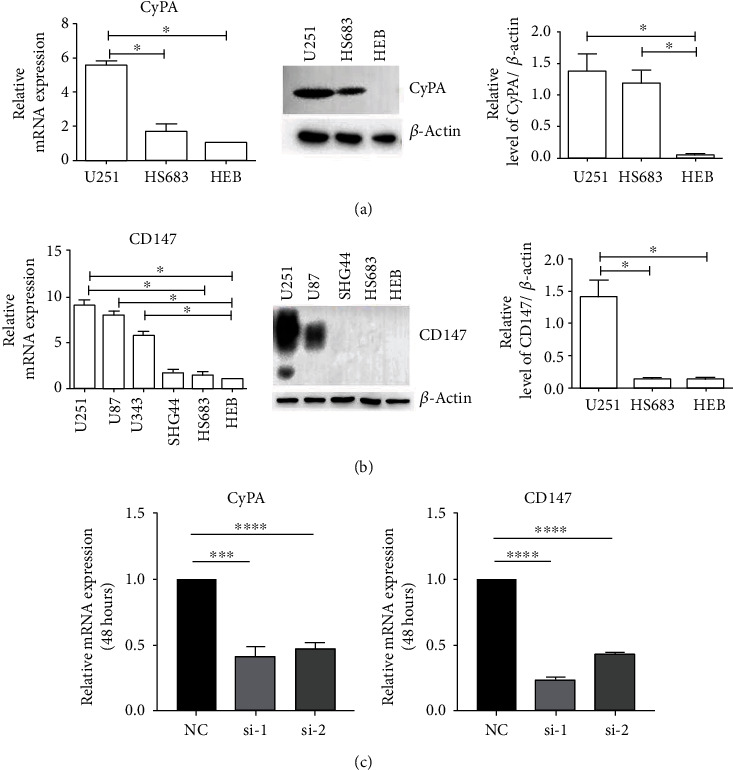
Expression and knockdown of CyPA and CD147 in glioma cell lines. The expressions of CyPA (a) and CD147 (b) in glioma cell lines are detected by RT-qPCR and western blot.(c) Specific si-RNA could inhibit CyPA and CD147 expression in U251 cell line. ^∗^*P* < 0.05, ^∗∗∗^*P* < 0.001, ^∗∗∗∗^*P* < 0.0001.

The expression of CyPA and CD147 in glioma cell lines is detected by RT-qPCR and western blot. CyPA is most highly expressed in U251 cell line compared with HS683 and HEB cell lines in mRNA level, which is further verified in protein level (Figure 2(a)). Similarly, the mRNA expression of CD147 is highest in U251 cell line compared with other cell lines, which matches the result from western blot analysis (Figure 2(b)). In order to explore the role of CyPA and CD147 in glioma, we utilize specific si-RNA to knockdown the expression of CyPA and CD147. Considering that the highest expression level is detected in U251 cell line, we transfect the specific si-RNA in U251 cell line whereas si-NC is transfected as the reference group. Results show that the cell morphology has not changed after the transfection (Figure S2). The expression of CyPA and CD147 is significantly inhibited after transfection for 48 hours (Figure 2(c)).

### 3.3. Knockdown of CyPA and CD147 Induces Glioma Apoptosis

**Figure 3:**
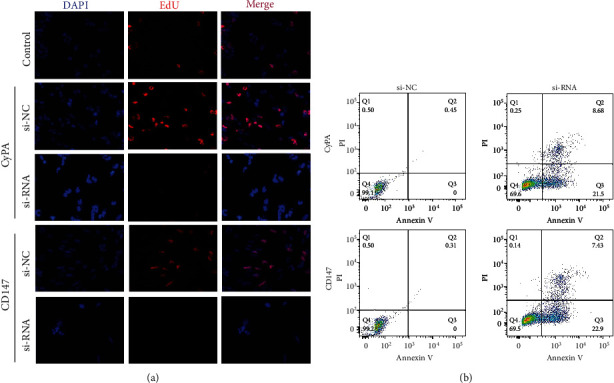
Knockdown of CyPA and CD147 reduces glioma proliferation. (a). EdU assay shows that inhibition of CyPA and CD147 significantly decreases glioma cell proliferation. U251 wild type cell is taken as the control group. (b). Flow cytometry analysis reveals the effect of CyPA and CD147 inhibition on glioma cell proliferation.

The rate of glioma proliferation is determined by EdU assay and flow cytometry analysis. U251 wild type cell line is taken as the control group in EdU assay. Results show that the blockade of CyPA and CD147 significantly reduces the proliferation of glioma cells compared with the si-NC group (Figure 3(a)). Flow cytometry analysis reveals that the transfection of si-RNA leads to a notable reduction of alive cells compared with the si-NC group (CyPA group: 69.6% vs 99.1%; CD147 group: 69.5% vs 99.2%) (Figure 3(b)). These results indicate that the inhibition of CyPA and CD147 may suppress cell proliferation by inducing cell apoptosis.

### 3.4. Prognostic Value of CyPA and CD147

**Figure 4:**
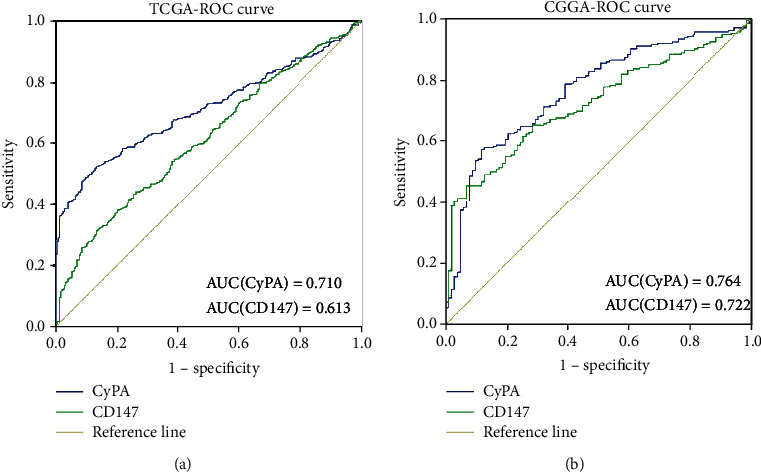
Predictive value of CyPA and CD147 in different grades of glioma. CyPA and CD147 expression pattern in different grades of glioma in TCGA database (a) and CGGA database (b) analyzed by ROC curve analysis.

Receiver operating characteristic (ROC) curves were generated using CyPA and CD147 expressions and WHO grades of glioma to examine the predictive value of CyPA and CD147. Areas under curve (AUC) of CyPA and CD147 are 0.710 and 0.613 in TCGA database, respectively (Figure 4(a)). In CGGA database, AUC of CyPA and CD147 are 0.764 and 0.722, respectively (Figure 4(b)). Despite the fact that CyPA and CD147 have predictive value in distinguishing different grades of glioma, the expression of CyPA and CD147 is also related to the recurrence of glioma [Table S2]. However, their expressions have poor correlation with age, sex, and tumor site.

## 4. Discussion

Several studies have demonstrated that CyPA and CD147 are upregulated in cancer and involved in diverse pathological processes during cancer promotion [22, 23]. In the present study, we use clinical specimens and database analyses to determine the high expression levels of CyPA and CD147 in high-grade glioma. The high expression of CyPA and CD147 is related to poor prognosis and rapid proliferation of glioma.

CyPA was initially identified as the major intracellular target of the immunosuppressive drug, cyclosporin A (CsA), which has therapeutic activities in several cancers including glioma [24]. Although the mechanism of antitumor activity of CsA in glioma is unclear, CsA can bind to CyPA and subsequently increase the chemotherapeutic effects of cisplatin in GBM [25]. The high expression of CyPA was frequently deemed as a biomarker in various cancers, which is consistent with our results. Despite being an intracellular protein, the extracellular CyPA (eCyPA) was demonstrated playing a critical role in a multitude of cancers [4]. CD147, also known as extracellular MMP inducer, is a membrane-bound glycoprotein identified as the principal receptor of eCyPA [26]. Recently, the expression of CD147 has been widely correlated with the progression of glioma [27]. Through cell-cell and cell-matrix interactions, CD147 induces expression of the matrix metalloproteins which are required for cancer invasion and metastasis [28]. As shown in Figure 1(b), the mean expression level of CD147 is higher in glioma than normal tissue although with no significant difference. This is mainly because CD147 is a widely expressed protein in many cell types including hematopoietic, epithelial, and endothelial cells [29]. Therefore, the expression level of CD147 is not significantly elevated in cancer development.

When performing the experiments, we take the methods of Mu et al. as the reference because we are investigating a similar field related to glioma. The major processes of cell culture, western blot, IHC, and RNA extraction are similar. We followed their methods as the protocols; however, the sequence of primers and the source of antibodies are not the same.

To better understand the impacts of genetic expression of tumor on clinical prognosis, TCGA was established to categorize genomic abnormalities in large populations around the world [30]. The convenient access to TCGA allows for large-scale gene expression profiling and database mining for potential correlation between genes and overall survival of glioma. However, TCGA database has a limitation; most specimens there are extracted from white or black people so that the gene profile of Asian patients could not be detailed explicitly. Hence, we further perform the analyses using CGGA database, in which most samples are acquired from Asian people. Multiple studies have used CGGA database in analyzing the correlation between gene expression and clinical features [31–33]. Because of the rapid development of whole-genome sequencing techniques, high-throughput tumor databases are gradually developed and become freely available to researchers. Large-scale database analysis would be an indispensable part in future researches.

Our study verifies that the high expression of CyPA and CD147 contributes to the proliferation of gliomas. Previous studies have shown that the interaction between CyPA and CD147 induces tumor invasion and proliferation [34, 35], indicating that CyPA and CD147 could be potential targets in cancer treatment. Currently, rare studies perform the inhibition strategy targeting CyPA during in vivo experiments whereas CD147 antibody is under evaluation in cancer treatment. The inhibition of CD147 by specific monoclonal antibody could induce cell death by impairing glycolytic energy metabolism in colon cancer and melanoma [36]. Moreover, CD147 inhibition was found to be of therapeutic benefit in an in vivo model of hepatocellular carcinoma and head and neck cancer [37, 38]. A randomized clinical trial indicates that the administration of CD147 antibody could significantly prevent hepatoma recurrence after live transplantation [39]. However, because of the broad pattern of CD147 expression, unintended side effects should be carefully assessed.

Glioma, especially high-grade glioma, is known for its poor prognosis. The age-standardized 10-year survival rate in low-grade glioma was 47% with median survival of 11.6 years [40, 41]. As for high-grade glioma, the median overall survival of anaplastic astrocytomas (WHO grade III) is approximately 3 years, whereas glioblastoma multiforme (WHO grade IV) has a poor median overall survival of 15 months [42]. However, the classification of gliomas remains a challenge before surgery. Liquid biopsy using cerebrospinal fluid (CSF) is promoted as a promising strategy providing cancer information with minimal injury. Tumor-derived DNA could be detected in CSF, and the genomic landscape of glioma in CSF is closely similar to genomes of tumor biopsies [43]. Given that the expression of CyPA and CD147 is significantly different in different grades of glioma and related to glioma recurrence, we believe that CyPA and CD147 could be potential biomarkers facilitating glioma categorization before surgery and further indicating prognosis.

## 5. Conclusions

In conclusion, our studies revealed that the different expressions of CyPA and CD147 axis could be potential biomarkers to indicate different grades of glioma and subsequently predict prognosis. Moreover, the downregulation of CyPA and CD147 induces cell apoptosis and inhibits cell aggressiveness, indicating that the axis could be a potential therapeutic target in glioma.

## Data Availability

The data used to support the findings of this study are included within the supplementary information files.

## Supplementary Materials

Supplementary 1 Figure S1: CyPA and CD147 expressions in different types of glioma specimens. The expression of CyPA (A) and CD147 (B) is detected in different types of glioma. Different grade of each histological type of glioma is defined according to WHO categorizations. The expression level of CyPA (C) and CD147 (D) is determined using mean density analysis. ^∗∗∗^*P* < 0.001, ^∗∗∗∗^*P* < 0.0001, ns stands for no significance.

Supplementary 2 Figure S2: cell morphology of U251 cell lines. The morphology of U251 is detected after the transfection of si-RNA.

Supplementary 3 Table S1: expression of CyPA and CD147 in clinical specimens. Table S2: correlations of CyPA and CD147 with clinical features of patients with glioma.
