# Supplementary material for: Downregulation of CyclophilinA/CD147 Axis Induces Cell Apoptosis and Inhibits Glioma Aggressiveness
Source: Biomed Res Int. 2020 Jul 24;2020:7035847. doi: 10.1155/2020/7035847 (PMC7396009; doi:10.1155/2020/7035847)
Supplement: Supplementary 3 — Table S1: expression of CyPA and CD147 in clinical specimens. Table S2: correlations of CyPA and CD147 with clinical features of patients with glioma. [file 7035847.f3.docx]

| Table S1 Expression of CyPA and CD147 in clinical specimens | | | | | | |
| --- | --- | --- | --- | --- | --- | --- |
| Sample type | WHO grade | Total | CD147 | | CyPA | |
|  |  |  | Positive | Ratio | Positive | Ratio |
| Normal tissue | N/A | 1 | 0 | 0 | 0 | 0 |
| Glioma | N/A | 21 | 10 | 47.60% | 9 | 42.90% |
| Pilocytic astrocytoma | I | 1 | 0 | 0 | 0 | 0 |
| Oligodendroglioma | II | 1 | 0 | 0 | 0 | 0 |
| Diffuse astrocytoma | II | 8 | 2 | 25.00% | 1 | 12.50% |
| Anaplastic astrocytoma | III | 6 | 4 | 66.70% | 5 | 83.30% |
| Glioblastoma | IV | 5 | 4 | 80.00% | 3 | 60.00% |

| Table S2 Correlations of CyPA and CD147 with clinical features of patients with glioma | | | | | | | |
| --- | --- | --- | --- | --- | --- | --- | --- |
| Features | | CyPA | | P value | CD147 | | P value |
|  |  | High (n=9) | Low (n=12) |  | High (n=10) | Low (n=11) |  |
| Age(y) |  |  |  | 0.748 |  |  | 0.223 |
|  | ≥55 | 2 | 2 |  | 3 | 1 |  |
|  | ＜55 | 7 | 10 |  | 7 | 10 |  |
| Sex |  |  |  | 0.528 |  |  | 0.505 |
|  | Male | 5 | 5 |  | 4 | 6 |  |
|  | Female | 4 | 7 |  | 6 | 5 |  |
| WHO grade |  |  |  | 0.011 |  |  | 0.049 |
|  | Ⅱ | 1 | 9 |  | 2 | 8 |  |
|  | Ⅲ | 5 | 1 |  | 4 | 2 |  |
|  | Ⅳ | 3 | 2 |  | 4 | 1 |  |
| Tumor site |  |  |  | 0.845 |  |  | 0.593 |
|  | Frontal | 5 | 6 |  | 6 | 5 |  |
|  | Temporal | 2 | 4 |  | 3 | 3 |  |
|  | Others | 2 | 2 |  | 1 | 3 |  |
| Recurrence |  |  |  | 0.017 |  |  | 0.005 |
|  | Yes | 7 | 3 |  | 8 | 2 |  |
|  | No | 2 | 9 |  | 2 | 9 |  |
